# Geriatrischer Patient mit neurologischer Symptomatik und verlängerter aPTT

**DOI:** 10.1007/s00108-023-01581-3

**Published:** 2023-09-20

**Authors:** Aisuluu Atakanova, Anne Heiligers, Martin Kirschner, Cornelius Bollheimer, Susanne Fleig

**Affiliations:** 1 Medizinische Klinik VI Altersmedizin, Uniklinik RWTH Aachen Standort Franziskus, Morillenhang 27, 52074 Aachen, Deutschland; 2 Klinik für Hämatologie, Onkologie, Hämostaseologie und Stammzelltransplantation, Medizinische Klinik IV, Uniklinik RWTH Aachen, Aachen, Deutschland

**Keywords:** Antiprothrombinantikörper, Antiphospholipidantikörpersyndrom, Polyneuropathie, Lupusantikoagulans, Thrombozytopenie, Anti-prothrombin antibodies, Antiphospholipid antibody syndrome, Polyneuropathy, Lupus anticoagulans, Thrombocytopenia

## Abstract

Ein 73-jähriger Mann mit vorbekannter demenzieller Entwicklung wurde mit Hypernatriämie bei Volumendepletion aufgenommen. Ursächlich dafür zeigte sich eine neue neurogene Schluckstörung, bei Z. n. mehreren Schlaganfällen bestand eine Halbseitensymptomatik. Bei der Sichtung der Unterlagen vorangegangener Krankenhausaufenthalte fiel wiederholt eine verlängerte aPTT (aktivierte partielle Thromboplastinzeit) auf; bereits fünf Jahre zuvor bestand ambulant der Verdacht auf ein Antiphospholipidantikörpersyndrom (APS), ohne dass die Diagnostik komplettiert worden wäre. Wir haben die Diagnose eines primären APS gestellt und eine Antikoagulation mit Vitamin-K-Antagonisten und ASS (Acetylsalicylsäure) begonnen.

## Abstract (en)

A 73-year-old man with dementia was referred to our clinic with hypernatremia and volume depletion. New-onset neurogenic dysphagia was likely the reason for both. The patient had chronic embolic strokes on the computed tomography (CT) images. Documentation from previous hospitalizations in different hospitals revealed a repeatedly prolonged activated partial thromboplastin time (aPTT); 5 years prior, antiphospholipid antibody syndrome had already been suspected, but the necessary workup was never completed. We diagnosed the patient with primary antiphospholipid antibody syndrome and initiated therapy with vitamin K antagonists (phenprocoumon) and aspirin.

## Anamnese, Untersuchung und Diagnostik

Ein 73-jähriger Mann wurde wegen einer seit einer Woche zunehmenden Vigilanzminderung geriatrisch stationär aufgenommen. Im Aufnahmelabor zeigte sich bei Exsikkose eine schwere Hypernatriämie mit 177 mmol/l [136–145 mmol/l], eine Thrombozytopenie (128/nl [150–400/nl]) sowie eine verlängerte partielle Thromboplastinzeit (aPTT) von 81,3 s (25–36 s).

Im kranialen CT (cCT) zeigte sich kein frischer Infarkt, aber im Vergleich zur Voruntersuchung waren postembolische Defekte rechts frontal, links parietal und links zerebellär sichtbar; zudem eine fortgeschrittene Hirnatrophie und eine ausgeprägte Leukenzephalopathie.

## Vorgeschichte

Mit 67 Jahren hatte sich der Patient wegen zunehmender Schmerzen und Parästhesien an den Extremitäten erstmalig neurologisch vorgestellt. Damals wurden eine axonal-demyelinisierende Polyneuropathie unklarer Genese und eine leichte bis mittelgradige kognitive Störung diagnostiziert. Bereits damals waren die aPTT-Werte erhöht (zwischen 58,9 und 72 s). Die weitere Diagnostik zeigte ein positives Lupusantikoagulans sowie erhöhte Prothrombinantikörper, sodass ein hochgradiger Verdacht auf ein Antiphospholipidsyndrom (APS) bestand. Kutane Veränderungen, typische thrombotische Ereignisse oder eine renale Beteiligung waren zu diesem Zeitpunkt nicht beschrieben.

Eine erneute laborchemische Kontrolle der Antikörper zur Diagnosesicherung nach den Sapporo-Kriterien sollte drei Monate später erfolgen. Der geplante Verlaufstermin wurde durch den Patienten versäumt, sodass die Diagnose nicht gesichert werden konnte.

In den folgenden fünf Jahren erfolgten mehrere ambulante neurologische Vorstellungen wegen Gangstörung, desorganisierten Verhaltens und kognitiver Defizite. Zehn Monate vor dem Aufenthalt bei uns war der Patient neurologisch stationär wegen demenzieller Entwicklung und schlechterem Gangbild; erneut war die aPTT spontan verlängert (49–56 s) ohne eine dies erklärende Medikation. Zwei Monate später erfolgte ein stationärer Aufenthalt mit Polytrauma nach Treppensturz, auch hier konnte eine verlängerte aPTT nachgewiesen werden. Zudem zeigten sich im cCT Zeichen einer Leukenzephalopathie sowie einer allgemeinen Hirnvolumenminderung.

Weitere vier Monate später kam es zu einer plötzlichen Persönlichkeitsveränderung mit aggressiven Zügen, woraufhin der Patient bei fremdaggressivem Verhalten psychiatrisch aufgenommen wurde und mehrere Monate stationär war; ein cCT aus diesem Aufenthalt ist uns nicht bekannt. Erst wenige Wochen vor der Aufnahme bei uns war der Patient in ein demenzsensibles Pflegeheim entlassen worden.

## Diagnose und Therapie

Wir wiederholten die Gerinnungsdiagnostik: Lupusantikoagulans (107 s [27–41 s], Lupus-RVVT-Verhältnis 2,0 [0,8–1,2]) und Anti-Prothrombin-IgG (66,7 U/ml [< 10 U/ml]) waren weiterhin positiv. Mit dem nachgewiesenen (älteren) embolischen Infarkt im cCT lag jetzt zudem ein thrombembolisches Ereignis vor, sodass nach Rücksprache mit der Hämostaseologie entsprechend den revidierten Sapporo-Kriterien [1] ein Antiphospholipidantikörpersyndrom sicher diagnostiziert und die Indikation zur therapeutischen Antikoagulation gestellt wurde. Es wurden bei hohem Risiko eine Antikoagulation mit Phenprocoumon (Ziel-INR 2,5–3) sowie eine gleichzeitige Plättchenhemmung mit ASS begonnen.

## Verlauf

Nach langsamer, kontrollierter Senkung der Hypernatriämie besserten sich die neurologischen Symptome nicht signifikant. Im stationären Verlauf zeigte sich eine ausgeprägte Schluckstörung und es kam konsekutiv zu einer schweren Aspirationspneumonie, die antiinfektiv behandelt wurde. In der Videoendoskopie bestätigte sich eine schwere neurogene Schluckstörung, sodass eine PEG-Sonde angelegt wurde. Fünf Monate nach der Entlassung hat der Patient eine persistierende Armparese, die PEG-Sonde konnte jedoch nach logopädischer Therapie mit Verbesserung des Schluckakts entfernt werden.

## Diskussion

Das Antiphospholipidsyndrom (APS) ist eine systemische Autoimmunerkrankung, die durch rezidivierende thrombotische Ereignisse in Patienten mit Antikörpern gegen Phospholipide bzw. assoziierte Glykoproteine (Anti‑β_2_-Glykoprotein 1, Anti-Cardiolipin-Antikörper oder positives Lupusantikoagulans) gekennzeichnet ist [1, 2]. Es manifestiert sich meist durch venöse (z. B. tiefe Venenthrombose oder Lungenembolie), arterielle (z. B. Schlaganfall oder TIA) oder mikrovaskuläre Thrombosen (jedes mikrovaskuläre Stromgebiet ist möglich). Nichtthrombotische Manifestationen sind z. B. Herzklappenerkrankungen, Livedo reticularis, Nierenschädigung durch thrombotische Mikroangiopathie oder chronische vasookklusive Läsionen, Thrombozytopenie, hämolytische Anämie und neue kognitive Einschränkungen [2]. Bei Frauen im gebärfähigen Alter manifestiert sich ein APS oft mit wiederholten Aborten nach der 10. Schwangerschaftswoche [3]. Das mittlere Alter zu Beginn der auf die Krankheit zurückzuführenden Symptome liegt bei 32 Jahren [3]; Erstmanifestationen im höheren Alter sind selten [4, 5]. Während in jungen Studienkohorten vornehmlich Frauen [6] betroffen sind, sind es bei Erstmanifestationen im Alter häufiger Männer [5].

Das Hauptantigen der Autoantikörper ist das β_2_-Glykoprotein 1 (β_2_GPI) auf der Oberfläche der Endothelzellen. Die Antikörperbindung kann verschiedene Effekte haben: Aktivierung von Immunzellen (Neutrophile, Makrophagen, Monozyten) und z. B. Komplementaktivierung, Ausschüttung von „vascular endothelial growth factor“ (VEGF) und Expression (prothrombotischer) zellulärer Adhäsionsmoleküle wie „tissue factor“ oder E‑Selektin; Formation sog. extrazellulärer DNA-Netze („neutrophil/monocyte extracellular trap“ [NET/MET]); Aktivierung der Gerinnung über gesteigerte Expression von Glykoprotein IIb/IIIa oder reduzierte Fibrinolyse und reduzierte Aktivität von Protein C sowie in der Schwangerschaft durch Komplementaktivierung Interaktion mit bzw. Apoptose von Trophoblasten und Deziduazellen [2]. Die Aktivität der AP-Antikörper führt zu lokaler Inflammation, Gefäßschädigung, Thrombosen oder zu Schwangerschaftskomplikationen [2].

Das Lupusantikoagulans bestimmt die Abhängigkeit der Gerinnung von Phospholipiden und nicht direkt einen spezifischen AP-Antikörper; somit kann das Lupusantikoagulans indirekt auch auf das Vorhandensein seltener Antikörper hinweisen (wie bei unserem Patienten die Anti-Prothrombin-Antikörper). Zunächst wird im Screening die aktivierte partielle Thromboplastinzeit (aPTT) als phospholipidabhängiger Gerinnungsparameter bestimmt. Bei verlängerter aPTT wird gemessen, ob sich nach 1:1-Mischung mit gepooltem Plasma die Gerinnungszeit normalisiert; dies würde z. B. auf einen Gerinnungsfaktormangel hinweisen. Korrigiert sich die Gerinnungszeit nicht, werden im dritten Schritt Phospholipide hinzugegeben. Verkürzt sich dadurch die Gerinnungszeit, ist das Lupuskoagulans bestätigt [2]. Häufig wird eine „ratio“ angegeben (Gerinnungszeit vor/nach Zugabe von Phospholipiden).

Nicht immer ist das Vorhandensein von AP-Antikörpern pathogen: in 10 % der Blutspenden (anamnestisch gesunder Spender) sind im Labor Anti-Cardiolipin-Antikörper nachweisbar, und ein positives Lupusantikoagulans in etwa 1 % [7]. Meist sind diese Werte allerdings nach einem Jahr nicht mehr nachweisbar, daher zählt der longitudinale Verlauf. Transiente Expression kann z. B. im Rahmen von Infektionen auftreten; auch deswegen müssen auffällige Laborparameter nach mindestens 12 Wochen wiederholt werden.

Das APS ist mit anderen Autoimmunerkrankungen vergesellschaftet und tritt häufig mit dem systemischem Lupus erythematodes (SLE) auf [1].

2006 wurden in einem internationalem Konsensusstatement die revidierten Sapporo-Kriterien als Diagnosekriterien für das APS veröffentlicht [1]. Ein APS liegt vor, wenn mindestens ein klinisches und ein laborchemisches Kriterium erfüllt sind: Klinische Kriterien sind der Nachweis einer arteriellen oder venösen Thrombose oder mikrovaskulärer thrombotischer Ereignisse, oder Schwangerschaftskomplikationen (näher erläutert in [1]); laborchemische Kriterien der Nachweis eines positiven Lupusantikoagulans, von Anti-Cardiolipin-IgG oder Anti‑β_2_GPI-IgG an zwei Zeitpunkten, die mindestens 12 Wochen auseinanderliegen [1]. Weitere klinische Manifestationen des APS wie Thrombozytopenie oder hämolytische Anämie sind bekannt, haben aber keinen Eingang in die diagnostischen Kriterien gefunden.

Eine spezifische Therapie existiert bis heute nicht. Zur Vermeidung neuer thrombembolischer und thrombotischer Ereignisse erfolgt eine therapeutische Antikoagulation [8]; hier scheint die Vitamin-K-Antagonisierung einen besseren Schutz zu bieten als ein DOAK [9, 10]. Bei Schwangeren würde man auf ein niedermolekulares Heparin ggf. kombiniert mit ASS umsteigen.

Im aktuellen Fall war die Diagnosestellung sowie die Indikation zur Therapie sehr verzögert: Die Latenz zwischen erstem Test und Bestätigung lag bei 5 Jahren, aufgrund eines versäumten ambulanten Termins. Neben den auffälligen Gerinnungsparametern und dem Nachweis der Anti-Prothrombin-Antikörper waren bereits damals eine Thrombozytopenie sowie die beschriebene neurologische Symptomatik auffällig; ein „klassisches“ thrombotisches Ereignis war zu diesem Zeitpunkt noch nicht bekannt, ebenso waren keine renalen oder kutanen Veränderungen beschrieben. Durch aktive erneute Einbestellung hätte die Diagnose aber bereits vor Eintritt der schweren Komplikationen gestellt werden können, was eine konsequente Antikoagulation nach dem ersten Ereignis ermöglicht hätte. Ebenfalls hätte eine digitale Patientenakte die Diagnose beschleunigt – der Patient war über die Jahre an mehreren Krankenhäusern und in mehreren Praxen, die kein verbundenes Befundsystem hatten, sodass die ausstehende Diagnostik nicht auffiel.

Im aktuellen Fall stand immer die neurologische Symptomatik im Vordergrund. Initial fiel eine axonal demyelinisierende Polyneuropathie auf. Eine Beteiligung der peripheren Nerven bei primärem APS kann eine Folge von ischämischer Thrombose der Vasa nervorum, Vaskulitis oder direkter Nervenschädigung durch pathogene Antikörper sein [11]. Kognitive Einschränkungen werden bei 42–80 % der Patienten mit primärem APS beschrieben [12].

Wir beschreiben die Erstdiagnose eines APS bei einem geriatrischen Patienten mit primär neurologischen Symptomen. Bei „grundlos“ verlängerter aPTT ist das APS eine wichtige Differenzialdiagnose, auch im Alter. Ein hohes Alter, zerebrale Läsionen und ein dreifach erhöhter Antiphospholipidantikörper zählen als Risikofaktoren für auftretende psychiatrische Symptome [12]; unser Patient war vor der Episode mit der Hypernatriämie, die ihn in unsere Behandlung brachte, über Monate in rein psychiatrischer Therapie gewesen.

## Fazit für die Praxis

- Bei wiederholt verlängerter aPTT ohne klare Ursache sollte ein APS abgeklärt werden.
- Höheres Alter und zerebrale Läsionen sind Risikofaktoren für psychiatrische Symptome eines APS.
